# Expanding the Curriculum in a School of Public Health

**DOI:** 10.3389/fpubh.2021.700638

**Published:** 2021-08-16

**Authors:** Evan J. Jordan, Sarah J. Young, Nir Menachemi

**Affiliations:** ^1^Department of Health and Wellness Design, School of Public Health, Indiana University, Bloomington, IN, United States; ^2^Department of Health Policy and Management, Richard M. Fairbanks School of Public Health, Indiana University Purdue University, Indianapolis, IN, United States

**Keywords:** curriculum, public health education, administration, education for public health, leisure and health

## Abstract

Public health education has long been concentrated in a core set of public health disciplines such as epidemiology, biostatistics, and environmental health. Despite leaps forward in our understanding of the myriad influences on public health, little has changed in the organization of our educational systems. One issue brought to the forefront of public consciousness by the COVID-19 pandemic is the importance of leisure experiences, such as nature walks, to mental and physical well-being. In this descriptive best practice article, we discuss our approach to expanding the notion of a school of public health and provide examples of how disciplines and subjects outside of the “norms” of public health education, including leisure studies, can help better prepare students for their future in the field. Leisure studies is just one of many subject areas that can add value to public health pedagogy, and we envision many other subject areas and departments integrating into schools of public health in the future.

## Introduction

It is recognized that in order to be maximally effective, public health scientists and practitioners must engage in greater multidisciplinary work with each other and with experts from outside disciplines not traditionally considered as core knowledge areas of public health (1). Virtually all current and future public health challenges (e.g., pandemic response, obesity, infant mortality, disparities, substance use disorders, health effects of climate change) require multidisciplinary partnerships to improve the understanding and ability to intervene successfully in assuring the health of populations. In support of this need, almost all federal, quasi-governmental, and private funders of public health work have made explicit calls for more multidisciplinary research and practice.

Yet despite these trends, an examination of departments contained within US schools of public health suggests that most institutions are organized under department names most closely aligned with the traditional disciplines of epidemiology, health behavior, health administration, environmental health, and biostatistics. Moreover, recent data suggests that doctoral students training in these disciplines rarely receive interdisciplinary experiences as part of their research activities; and when they do, it is typically between two public health disciplines (e.g., epidemiology and biostatistics) rather than between a public health disciplines and a different scientific field (e.g., nutrition, economics, genetics) (2).

Within the practice community, myriad success stories exist where multidisciplinary partnerships including public-private ones, have resulted in improvements to community and population health. In Dallas, TX, a large health system partnered with the local park and recreation department to create a primary care clinic that integrates wellness and prevention programs into a city recreational center (3). Use of the clinic was associated with a reduction in emergency and hospital utilization as well as the associated costs of these services. In Louisville, KY, a technology firm partnered with the metro government and a non-profit institute to equip asthma inhalers with sensors to identify exact locations where rescue inhalers are most often used within the city (4). This information, when combined with environmental data, led to a significant reduction of asthma burden following policy interventions to enhance tree canopies, targeted air pollution abatement strategies, and recommend new truck routes. Separately, several leading organizations have developed innovative approaches to engaging patients by revamping the consumer experience in health care (5). By addressing patients' unmet information and communication needs, and/or by addressing desires expressed by patients (e.g., to have some control over their physical environment within the hospital) health care systems in Louisiana and California have demonstrated the benefits to many stakeholders that could be accomplished with interdisciplinary work.

In all of these cases, diverse experts with training in, for lack of a better term, “leisure” disciplines (including parks, recreation, hospitality, tourism, and events) worked closely with public health researchers and practitioners in a way that models the mindset and skillset needed by the future public health workforce. Indeed, there are many more opportunities for experts in hospitality management (6–8) and parks and recreation (9–12) to enhance the work of public health. However, public health students have traditionally not been exposed to the theories, practices, nor methods of these disciplines.

The purpose of the current paper is 3-fold. First, we aim to espouse the benefits of including non-traditional disciplines in schools of public health. Second, we describe the recent integration of the former Department of Recreation, Parks, and Tourism Studies in the School of Public Health at Indiana University as the newly imagined Department of Health and Wellness Design as a framework for schools wishing to expand the public health curriculum to include non-traditional disciplines in the future. Third, we discuss the integration of a leisure-based curriculum into a School of Public Health and discuss how it is both similar and different from traditional schools of public health.

## Benefits of Inclusion

Important work in many areas of academic inquiry is currently occurring that, while indeed addressing public health issues, may not be viewed as such by traditional public health disciplines as evidenced in academic papers, university marketing, or in the classroom. In our case, we believe that the academic pursuits in the leisure disciplines are *entirely* about public health. One factor made painfully obvious by the recent COVID-19 pandemic is the importance of leisure pursuits to the health of individuals and populations. Despite the many social distancing and shelter in place restrictions imposed on individuals across the globe—public health experts have encouraged the public to continue to recreate—to take hikes in the woods, to stay active, to play games in their own back yards—all to help cope with the stress and grief of the pandemic. Beyond rare pandemic conditions, imagine a world without public parks where city dwellers could have a picnic, no areas of protected wilderness in which to hike, and no hotel spas available for an afternoon of pampering. These are all community resources that have been linked to improved health and well-being of those who use them (13). In these domains and more, we believe the mindset and skillset necessary to promote healthy behaviors is somewhat different than that of traditional public health disciplines.

We believe that educators from a leisure background, compared to traditional public health backgrounds, are more likely to stress the promotion of enjoyable healthy behaviors as opposed to discouraging unhealthy ones. To be sure, both are necessary; but this is one approach that can perhaps help change the perceptions of the public about being healthy in general. Through research based on theories of psychology (14), sociology (15), business (16), environmental education (17), and more, leisure-based educators explore health from varying perspectives. Instead of educational messages about not smoking, cutting back on sugar, and adhering to prescribed medicines—-leisure-based educators focus on making sure people take time off from work, find time to relax, take outdoor hikes, plan overdue vacations, or enjoy time at the spa. Including outside disciplines has the potential to supplement, not *replace* the interventions utilized by traditional public health practitioners thus increasing the appeal (and success) of health programs among the general public. For example, taking a vacation has not been studied for its effect on diabetes (yet), but research has linked vacationing to improved wellness on a holistic level that transcends diseases (13). These benefits span from simply feeling happier and healthier (18), to reducing stress (19) to potentially having positive effects on the incidence of cardiovascular disease (20).

As funding agencies further require elements of inter- and multi-disciplinarity in their proposals, schools of public health will need the diversity in expertise that non-traditional public health disciplines can provide. Indeed, any school of public health that expands the notion of the type of education and research that are a part of the public health equation should have a significant advantage in the coming years with respect to successfully competing for extramural funding. Furthermore, the inclusion of a leisure based department in a school of public health serves to provide additional opportunities for students in traditional public health disciplines to work with professionals outside of tradition public health fields, one foundational competency for MPHs according to the Council on Education for Public health (CEPH) (21).

## A Roadmap for Inclusion

From its inception, the U.S. leisure movement has focused on the protection of open spaces and healthy recreational activity as a mean to promote health and well-being (22). Yet, it has not always been clear that a leisure-based curriculum fits in a traditional school of public health? For maximum impact, a natural evolution of two historically independent fields must take place.

The leisure studies literature has advocated for change and integration within the public health arena. Talmage et al. [(23), p. 28] plainly stated, “expansion and stretching are needed in the parks and recreation field rather than isolation and myopathy to attain broad, positive impact for the profession and society.” While others have suggested there is a place for the study of leisure in public health, scholars from positive psychology, education, public health and health promotion have lauded the concepts of “the need to reconnect to nature, mindfulness for stress relief, and more attention to the positive aspects of subjective experience in the interest of health and well-being” [(24), p. 13].

So, how does an established curriculum and academic department in leisure studies evolve for inclusion in a school of public health? First, a panel composed of administrators and faculty from within the department and school of public health studied enrollments, curriculum, grant generation, faculty content and research areas to determine strengths and weaknesses. This was followed by a similar evaluative process by an external panel composed of leisure scholars, public health scholars, and industry representatives from around the U.S. After analysis of the department and its metrics, it was recommended that the department be re-organized to leverage its strengths in the needed support of the public health mission. This called for a re-branding exercise and renaming of the department.

In re-inventing the department with a new vision and mission, existing faculty members were asked to answer the following questions:

- What is the concept of the department? What is the intellectual thread that our faculty share? How might this connect with faculty in other academic areas of the school of public health?
- Imagine if we were starting with a clean slate…what type of department involving recreation, parks, tourism, hospitality, event management, community engagement and health outcomes should be created?
- What meets the needs of society, existing service fields, and the desires of undergraduate students who are looking to start their career paths?
- What type of department could generate research and external funding to support research in those settings?

A Dream Statement Workshop (DSW), or a collective thinking approach using mind-mapping, was implemented to help develop a vision and mission for a new department. The DSW is a proprietary approach developed by our facilitator over many years of conducting such exercises. It included the collaboratively answering the following questions:

- Imagine an award-winning faculty member from our department in 2049. What do they look like?
- Imagine the first round of graduates of our new program—what does their future look like?
- What concepts do we need to focus on for our new department to be successful?
- What do the vision, mission, and name of this department look like?

Utilized in the business world to help companies develop new products, services, and marketing communications, it was believed that the DSW process could be adapted to help create and design a new organizational element fulfilling the departmental vision outlined by the faculty. In other words, we wanted to create a safe opportunity to ideate, define, and outline a new raison d'etre oriented around advancing public health through settings where people live, work, learn, and recreate.

**Figure 1:**
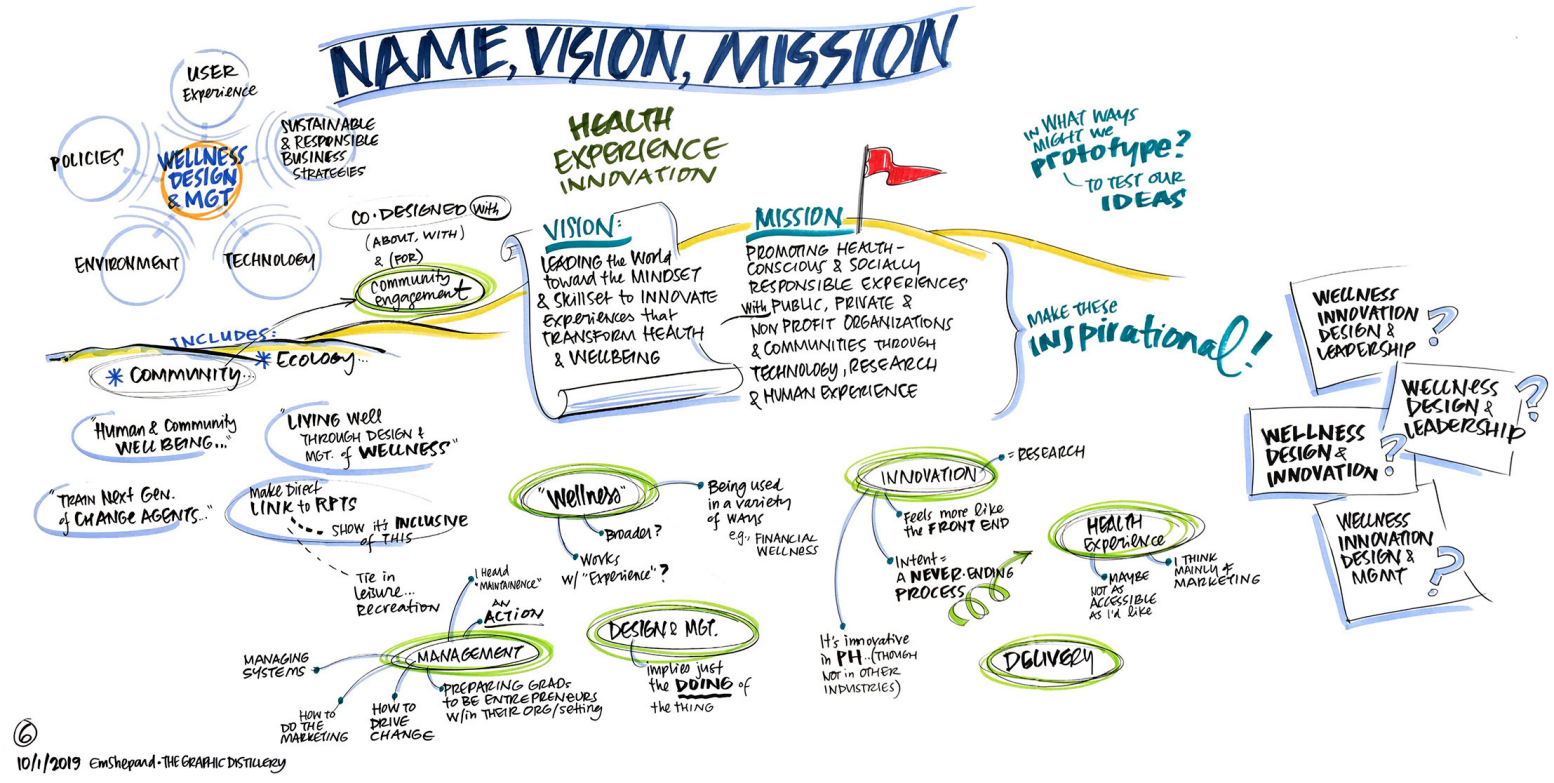
What do the vision, mission, and name of this department look like?

**Figure 2:**
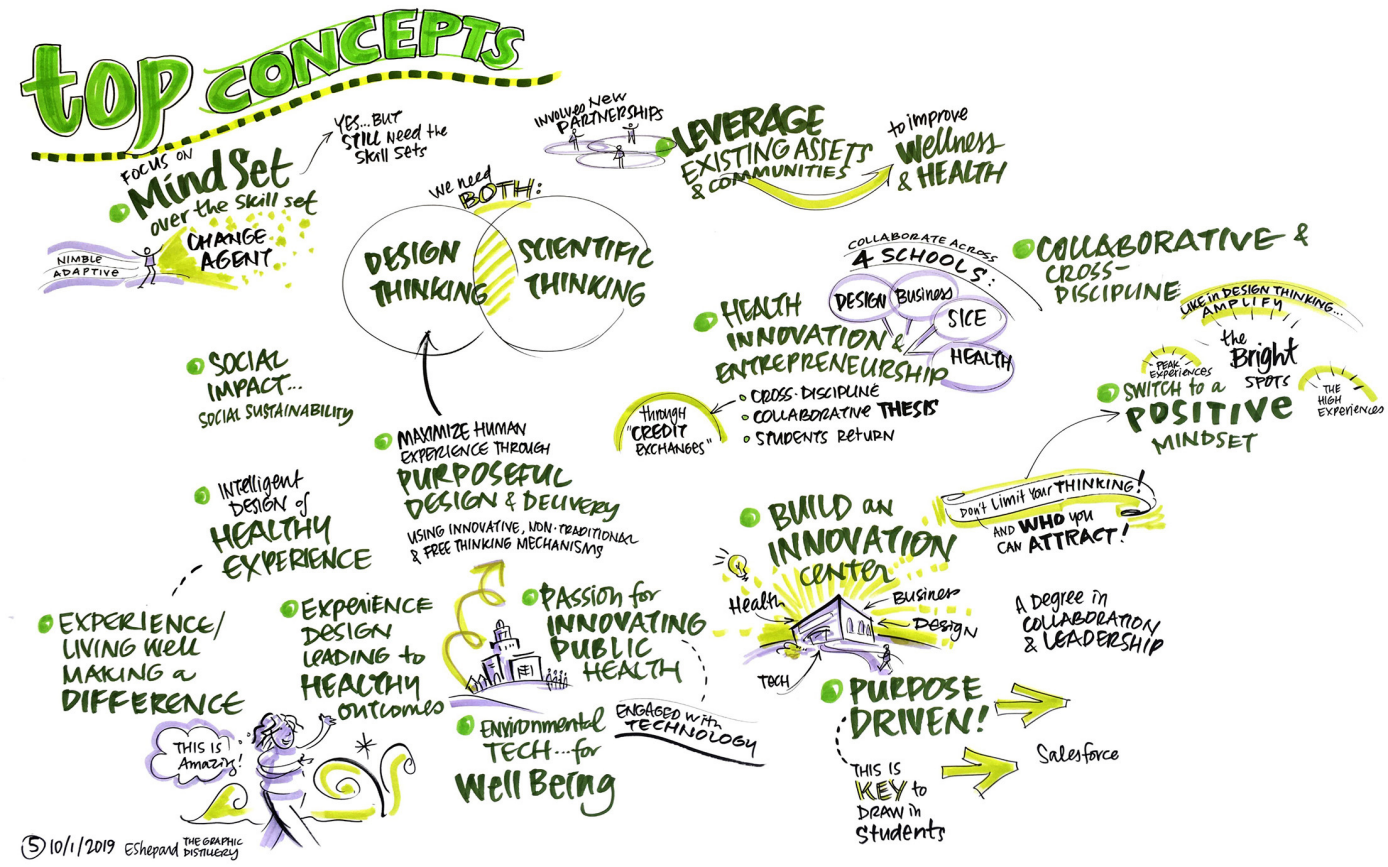
What concepts do we need to focus on for our new department to be successful?

**Figure 3:**
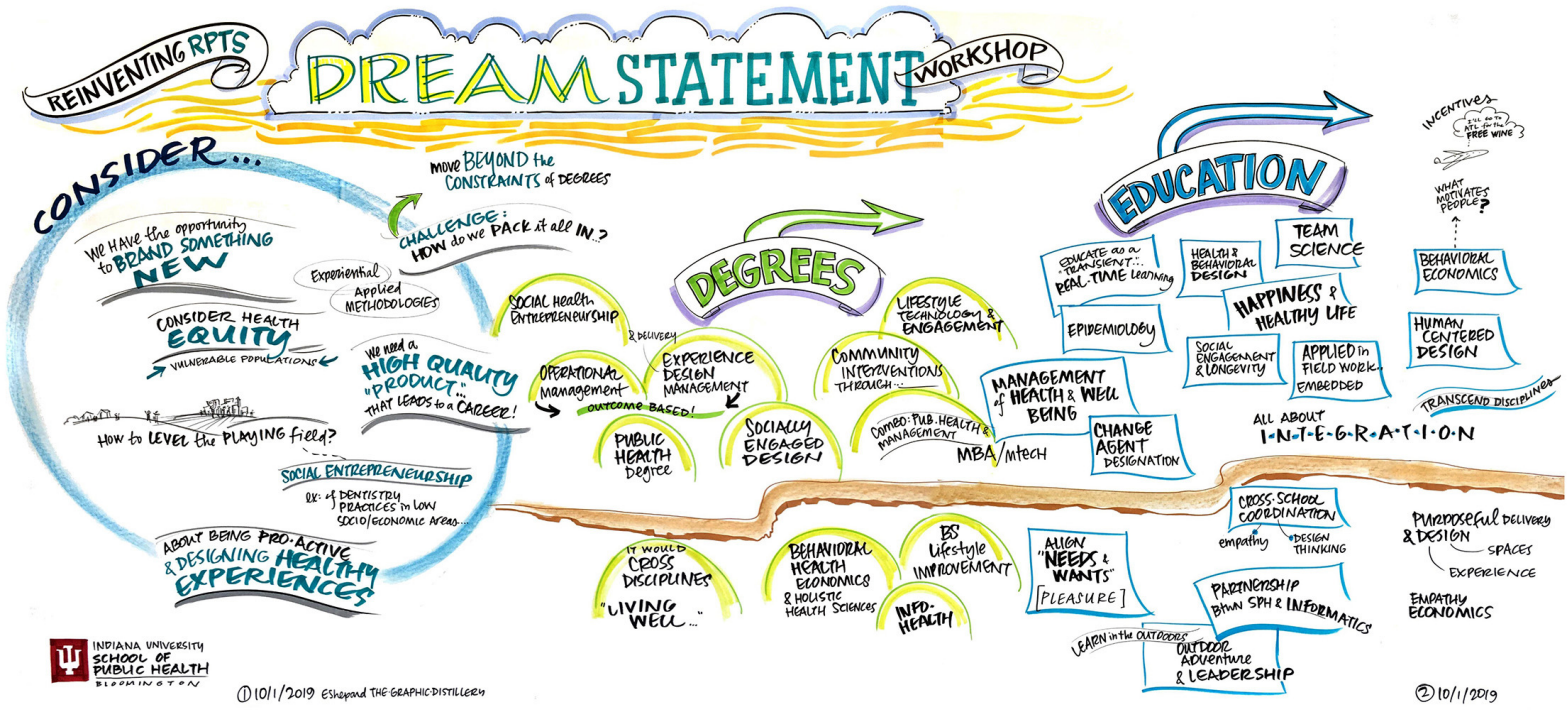
Imagine an award-winning faculty member from our department in 2049. What do they look like?

**Figure 4:**
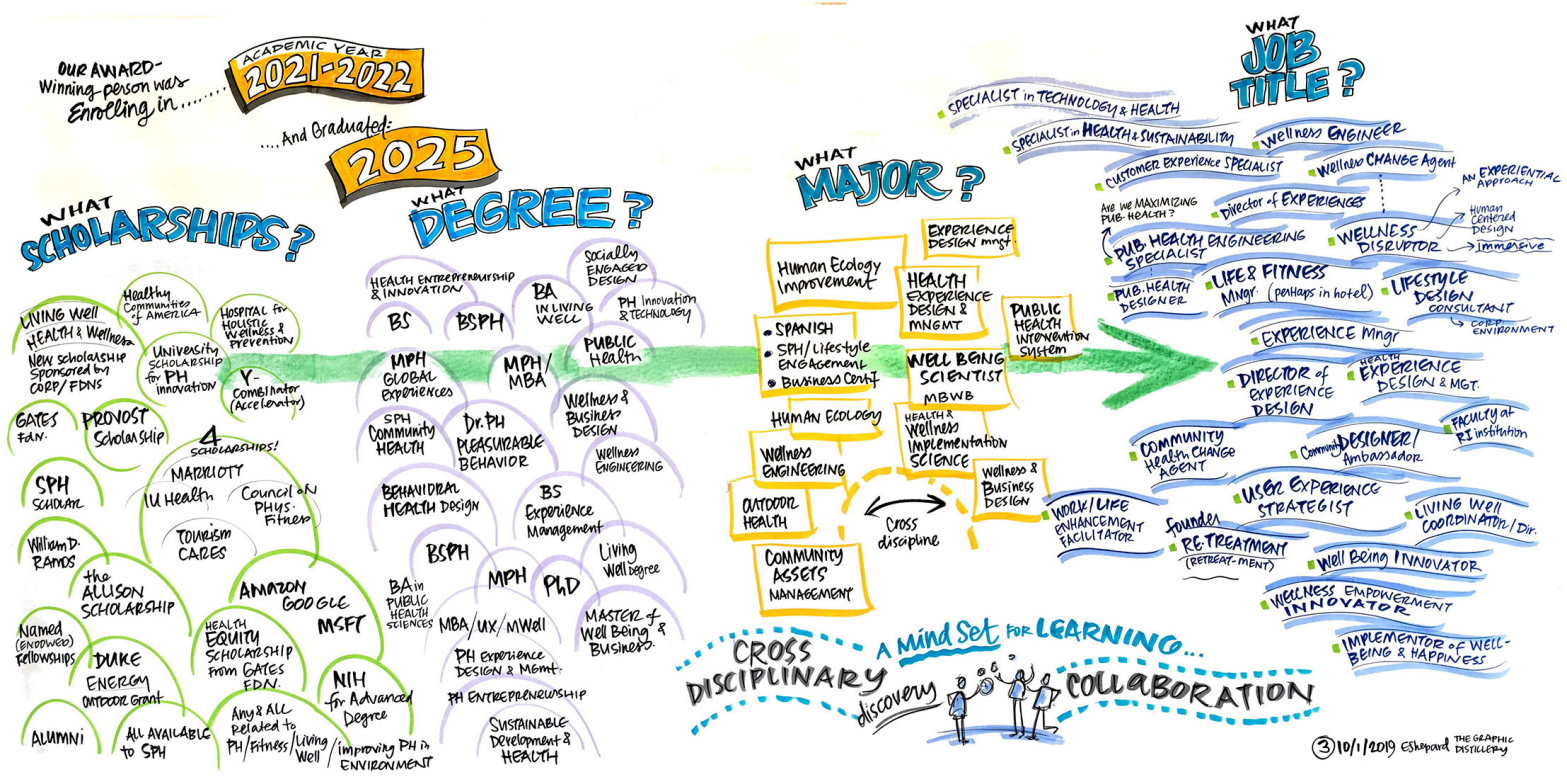
Imagine the first round of graduates of our new program - what does their future look like?

To ensure diverse stakeholder representation at the DSW, members of the current departmental faculty were joined by design experts, public health experts, administrators from the university enrollment management office, other school of public health faculty, and medical school faculty in a 1-day workshop. Additionally, a graphic facilitator was utilized in order harness the power of visuals in an effort to clarify and align participants' thinking, and to enable the collaboration needed for effective change (Figures 1–4).

The DSW utilized a modified backwards design (25) process relying upon the creativity and “dreaming” of its participants to imagine the desired results of future students graduating from the department. Brainstormed big ideas were recorded along with requisite assessments and activities necessary to make the desired results occur. At the end of the DSW, diverse viewpoints and ideas coalesced into a framework from which our faculty could build. Further discussions among the faculty solidified the framework, and feedback from current, former, and potential future students were solicited regarding possible new department names.

The Department of Health and Wellness Design ultimately emerged from the process with a mission of integrating and delivering health, happiness, and well-being in all organizations, activities, and experiences through research, education, and service. The faculty of this reimagined department are committed to studying various innovative approaches, methods, and strategies that enable optimal health for all people with equity, dignity, and impact. As health needs and challenges evolve, the power of designing and promoting healthy experiences to enhance human well-being has never been more relevant as in the 21st century.

## The Health and Wellness Design Curriculum

The Department of Health and Wellness Design is constantly evolving its curriculum to provide the best academic experience for our students. Our department has a total of 347 undergraduate students and 50 graduate students. Our students come from diverse geographical and demographic backgrounds. In the past, the curriculum of the former department of Recreation, Parks, and Tourism Studies had focused on training graduates for employment in the parks, recreation, and tourism industries. Upon shifting to our new home in the School of Public Health, changes to the curriculum at both the undergraduate and graduate levels occurred incrementally. In the following paragraphs we discuss how our curriculum has evolved as a result.

At the graduate level, all students are required to take a non-credit bearing onboarding course titled Foundational Knowledge in Public Health. This twelve-module course aligned with the 12 learning outcomes in foundational public health knowledge as defined by the Council on Education for Public Health (CEPH). Other changes included all Ph.D. students being required to take a course in epidemiology and an increased number of elective courses from other departments within the school earning credits toward departmental M.S. degrees. The department also created new graduate courses more closely related to public health such as those titled “Human Health, Quality of Life, and Natural Environments” and “Leisure as a Determinant of Health.” Lastly at the graduate level, a new MPH in Parks and Recreation was created and launched in Fall 2017 designed to educate students on public health approaches in the management of recreation services and resources in a wide variety of leisure settings.

Changes to the undergraduate curriculum were also made. In an introductory course called Foundations of Leisure and Public Health, public health core competencies were integrated into the student learning outcomes. Similar to changes made to the graduate curricula, undergraduate majors were also provided with more elective courses from other departments within the school that contributed to their degree requirements. Lastly, new courses within the department were created and integrated into curriculum offerings, such a course titled “Human Health and Natural Environments.” Beyond new courses, elements of public health were integrated into the majority of existing courses within the department, emphasizing the role that the leisure disciplines play in the health and well-being of both individuals and the population at large. The undergraduate core curriculum now reflects a joining of leisure and public health (Table 1).

**Table 1:** Department of health and wellness design undergraduate core curriculum.

**Course**	**Credits**
Foundations of leisure and public health	3
Inclusion in recreation, parks, and tourism	3
Management in recreation, parks, and tourism	3
Career and internship preparation	3
Data-based decision making	3
Event planning and program development	3
Professional internship	12

The department's graduate programs now consists of three degrees—an M.S., and M.P.H., and a Ph.D. The M.S. has three distinct focus areas—recreational therapy, tourism management, and parks, recreation, and outdoor management. The newly formed MPH focuses on the linkages between recreation and public health, providing the skills needed for planning, implementing, and evaluating creative leisure and recreation interventions for positive health outcomes. Finally, the updated Ph.D. program in leisure behavior emphasizes innovation and takes a multidisciplinary approach to the study of leisure behavior and its effects on physical and mental health. The undergraduate degree has four major focus areas: (1) Outdoor Recreation, Parks, and Human Ecology, (2) Public, Non-profit, and Community Recreation, (3) Recreational Therapy, and (4) Tourism, Hospitality, and Event Management. Each major focus area emphasizes the health and wellness benefits of engaging in their unique mix of leisure activities. The full departmental curriculum can be found at: https://publichealth.indiana.edu/research/departments/health-wellness-design/index.html.

### Similarities and Differences to Traditional Public Health Curriculum

The first and perhaps most important distinction between the Health and Wellness Design curriculum and that of other schools of public health is the inclusion of leisure as a major subject area. According to information obtained from the Association for Schools and Programs of Public Health (ASPPH), there are no other departments within a U.S. school of public health that focuses on a leisure-based curriculum. However, there are two existing degrees offered at other schools of public health that appear to overlap with our program. One, at Temple University, includes a B.S. and M.S. in Recreational Therapy, housed within the department of health and rehabilitation sciences. The second is at the Colorado School of Public Health, which offers an M.P.H. in Physical Activity and Healthy Lifestyles. While the Recreational Therapy curriculum at Temple University matches our Recreational Therapy curriculum closely, the MPH in Physical Activity and Healthy Lifestyles curriculum appears to include few courses with a leisure component.

## Conclusion

In this paper, we have discussed the benefits of expanding the curriculum of a school of public health, offered a roadmap for doing so based on our experience integrating the Department of Health and Wellness Design into the Indiana University Bloomington School of Public Health, and discussed the curricular changes that aided integration. In our examination of other US based schools of public health, it appears as though our expansion of public health discourse to include leisure-based topics is unique, although there are several other programs that do offer a limited number of recreation-based courses. We believe that our experience can serve as a template for the expansion of a school of public health curriculum that can be used by others considering similar transitions, including with other non-traditional public health disciplines in pursuit of improved public health education, research, and practice.

## Data Availability Statement

The original contributions presented in the study are included in the article/supplementary material, further inquiries can be directed to the corresponding author/s.

## Author Contributions

EJ, SY, and NM contributed substantively to the conceptualization and writing of this article. All authors contributed to the article and approved the submitted version.

## Conflict of Interest

The authors declare that the research was conducted in the absence of any commercial or financial relationships that could be construed as a potential conflict of interest.

## Publisher's Note

All claims expressed in this article are solely those of the authors and do not necessarily represent those of their affiliated organizations, or those of the publisher, the editors and the reviewers. Any product that may be evaluated in this article, or claim that may be made by its manufacturer, is not guaranteed or endorsed by the publisher.
